# The Change4Life Convenience Store Programme to Increase Retail Access to Fresh Fruit and Vegetables: A Mixed Methods Process Evaluation

**DOI:** 10.1371/journal.pone.0039431

**Published:** 2012-06-27

**Authors:** Jean Adams, Joel Halligan, Duika Burges Watson, Vicky Ryan, Linda Penn, Ashley J. Adamson, Martin White

**Affiliations:** 1 Institute of Health & Society, Newcastle University, Newcastle upon Tyne, United Kingdom; 2 School of Medicine and Health, Durham University, Stockton on Tees, United Kingdom; 3 Human Nutrition Research Centre, Newcastle University, Newcastle upon Tyne, United Kingdom; The University of Hong Kong, Hong Kong

## Abstract

**Background:**

Consumption of fruit and vegetables is important for health, but is often lower than recommended and tends to be socio-economically patterned with lower consumption in more deprived groups. In 2008, the English Department of Health introduced the Change4Life convenience store programme. This aimed to increase retail access to fresh fruit and vegetables in deprived, urban areas by providing existing convenience stores with a range of support and branded point-of-sale materials and equipment.

**Methods:**

We undertook a mixed-methods study of the Change4Life convenience store programme in the North East of England around two years after initial implementation. Store mapping (n = 87; 100% stores) and systematic in-store observations (n = 74; 85% stores) provided information on intervention fidelity; the variety, purchase price and quality of fresh fruit and vegetables on sale; and purchase price compared to a major supermarket. Ten qualitative interviews with a purposive sample of retailers and other professionals explored experiences of the intervention and provided further insight on quantitative results.

**Results:**

Intervention stores were primarily located in socio-economically disadvantaged areas. Fidelity, in terms of presence of branded materials and equipment, was low and much was not being used as intended. Fresh fruit and vegetables on sale were of high quality and had a purchase price around 10% more than comparable products at a major supermarket.

Interviewees were supportive of the health improvement aim of the intervention. Retailers were appreciative of part-funding for chill cabinets and free point-of-sale materials. The intervention suffered from: poor initial and on-going communication between the intervention delivery team and retailers; poor availability of replacement point-of-sale materials; and failure to cement intended links with health workers and community organisations.

**Conclusions:**

Overall, intervention fidelity was low and the intervention is unlikely to have had a substantial or long-term effect on customers’ consumption of fruit and vegetables.

## Introduction

Consumption of fruit and vegetables is important for health. [1] Promotion of fruit and vegetable consumption is a key public health strategy, underpinned by the ‘five-a-day’ message (adults should eat at least five 80 g portions of fruit and vegetables per day). [2] Yet average daily consumption among UK adults is just over two portions. [3] Fruit and vegetable consumption in the UK, as elsewhere, is strongly socio-economically patterned, with lower consumption among individuals living in more deprived circumstances. [3], [4]

There has been a recent focus on structural interventions to promote healthier diets, particularly in more deprived groups. For example, since 2004, the Scottish Grocers Federation Healthy Living Programme has promoted fresh fruit and vegetables (FFV), as well as other ‘healthier’ products, in convenience stores. [5] These initiatives are often based on the assumptions that convenient retail access to the components of a healthy diet increases consumption and that such access is worse in more deprived areas. [5] In the UK, neither of these assumptions are supported by robust evidence.

The term ‘food desert’ has been used to describe an urban area where it is difficult to buy the components of a healthy diet at a reasonable price. [6] A recent systematic review found that there is little evidence for the existence of food deserts in high income countries other than the USA. [7] Poor retail access to the components of a healthy diet is, therefore, unlikely to make a substantial contribution to unhealthy dietary patterns in the UK.

Few robust outcome evaluations of the effects of improved retail access to fruit and vegetables on dietary quality have been conducted. [8] A recent systematic review found that small food store interventions to improve diet may increase availability and sales of healthy foods, and customer knowledge, but that there is an absence of evidence of effect on individual diet or health. [8] Evaluation of the Scottish Grocers Federation Health Living Programme indicated that the intervention resulted in increased sales of FFV from participating stores. [9] But, this does not necessarily equate to increased FFV consumption.

Despite the limited evidence base in this area, in 2008, the English Department of Health (DH), working in partnership with the Association of Convenience Stores, embarked on an intervention to improve provision of FFV in convenience stores in deprived, urban areas in England with poor existing retail access to FFV. [10] This intervention was branded as part of Change4Life, the national obesity prevention programme, and was introduced sequentially across English regions starting in the North East region.

In the UK, convenience stores are defined as grocery stores selling food and drink for off-premises consumption as their main activity, with less than 3000 ft^2^ floor area, and open more than eight hours per day, seven days per week. [11] There were around 50,000 UK convenience stores in 2008, representing approximately 20% of the food and grocery market. [11]

We conducted a process evaluation of the Change4Life convenience store intervention in the North East of England.

## Methods

We performed a mixed-methods study using quantitative mapping and systematic in-store observations to provide information on intervention fidelity; the variety, purchase price and quality of FFV on sale; and purchase price compared to a major retailer. One-to-one qualitative interviews were conducted with a purposive sample of key stakeholders to explore their experiences of the intervention and provide further insight on quantitative results.

### Intervention Description

#### Intervention leadership and delivery

Department of Health civil servants provided strategic leadership for the intervention. Implementation was co-ordinated by a project delivery team. A national steering group consisted of the DH team, the project delivery team, and national representatives of each of the symbol groups involved (see below). A regional steering group was also established consisting of representatives of the DH leadership team, the project delivery team, regional symbol group managers, the regional obesity lead, and local health workers (see below).

#### Intervention stores

The UK convenience store sector includes: independent stores, petrol station forecourt stores, branches of multiple retailers, and members of symbol groups. Symbol group stores have common brand identity, and access wholesale goods via regional managers. All stores that took part in the intervention were members of symbol groups. Stores were selected for inclusion by consultation between the project delivery team and regional symbol group managers. It was intended that all stores should be located in socio-economically deprived, urban areas with poor existing retail access to FFV.

#### Intervention components

An intensive intervention (see Table 1) was introduced in 17 ‘demonstration’ stores in the North East region and a less intensive intervention (see Table 1) was provided to 70 further ‘roll-out’ stores between October 2008 and June 2009. After initial implementation, the regional steering group decided that each intervention store should also be linked to a local ‘health worker’ (public health professional employed by local primary care organisations) to help develop links to local schools and community initiatives to which intervention stores might be able to supply FFV.

**Table 1:** Summary of intervention models.

	Demonstration Stores	Roll-out stores
Number of stores	17	70
Target location	deprived, urban area with limited existing retail access to fresh fruit & vegetables	
Point-of-sale materials & equipment	provision of Change4Life branded materials, including: shelf ‘barkers’; shelf strips; A2 posters; chill stickers; window vinyls; price over-clips	
In-store support	appointment of an existing staff member as ‘Fresh Food Champion,’ to oversee: fresh fruit & vegetables orders and stocking, and compliance with Change4Life guidance	
Launch leaflet	launch leaflet in Healthy Start mailings, sent to households within a 1 mile radius	
Intervention support	extensive support from project delivery leader	
Training for store personnel	training DVD and manual	
Chill cabinet	Department of Health provided 50% of cost of anew chill cabinet dedicated to fresh fruit &vegetables	Change4Life branding materials provided for any existing chill cabinet
Store layout changes	significant changes, including: moving fresh fruit &vegetables to front of store; expanding spacededicated to fresh fruit & vegetables; mobile fresh fruit &vegetable stand	some changes, possibly including: moving fresh fruit & vegetables to a more prominent position; expanding space dedicated to fresh fruit & vegetables; mobile fresh fruit & vegetable stand
Promotional activities	in-store sampling of fresh fruit & vegetables by localchildren and support for integration with otherChange4Life campaigns, including Cook4Life andBreakfast4Life; with support from local Primary CareTrust	support for integration with other Change4Life initiatives, including Cook4Life and Breakfast4Life; with support from local Primary Care Trust
Average cost per store	£5100	£300

### Quantitative Research and Systematic In-store Observations

#### Inclusion criteria & recruitment

All 87 intervention stores in the North East of England were eligible to take part in the study. All stores were mapped based on the demographic profiles of areas. Stores were also sent a letter informing them that a researcher would visit their store to conduct observations within two months of receipt but that they could opt-out of visits if they wished.

#### Variables of interest & data collection

Fidelity of the intervention in terms of location in a deprived, urban area with poor existing retail access to FFV was explored using mapping. Postcodes of intervention stores were used to determine the lower super output area of location (small administrative areas with mean population of around 1500). [12] Routine data were then used to determine if stores were located in deprived (defined as the most deprived 40% of areas in England using the Index of Multiple Deprivation 2007 [13]), urban (defined as an urban area with population over 10,000 using National Statistics urban/rural classification [14]) areas with poor existing retail access to FFV (defined as located more than 500 m network distance from a supermarket).

Fidelity of the intervention in terms of intended use of Change4Life branded point-of-sales materials and equipment was explored using observations structured by a data collection proforma. We collected information on the presence or not of a Change4Life branded chill cabinet, shelve strips, and dedicated FFV stand and whether or not these were used appropriately (chill cabinet only to contain FFV or bottled water, with no potatoes, onions or bananas; shelf strips only on shelves containing FFV; mobile stand containing only FFV). Store personnel were asked if the store had a FFV Champion.

Variety, quality and purchase price of FFV were assessed using observations structured by the data collection proforma. For each FFV line (i.e. specific different types of products – royal gala apples, golden delicious apples, braeburn apples etc.) on sale, quality was assessed and judged to be poor if there was obvious evidence of skin damage, rot or mould, bruises or discolouration on any of the items on sale.

A sample of 10 stores was visited simultaneously by an additional researcher who conducted independent observations. Inter-rater agreement was above 75% for all items and above 95% for FFV availability, quality and purchase price. All in-store observations were conducted in February and March 2011.

Within five weeks of in-store observations, purchase prices of comparable items available from the largest UK supermarket were identified and recorded, using www.tesco.com. Mean purchase price per line as a proportion of purchase price at www.tesco.com for each store was calculated. As far as possible, products were matched for line, pack size and brand. Best available matches were substituted where exact product matches were not available.

### Qualitative Interviews

#### Inclusion criteria & recruitment

Members of two key stakeholder groups were selected to take part in the qualitative component: individual retailers, and other professionals involved in intervention leadership and delivery. Individual retailers were purposively sampled to reflect both demonstration and roll-out stores and recruited during in-store visits. Other professionals were purposively sampled to reflect those involved in leadership and delivery at a variety of levels and recruited via telephone calls to their workplace.

#### Data collection & topics of interest

Interviews were guided by a prospectively developed topic guide that covered interviewees’ motivation for taking part in the intervention; their views about barriers and enablers to intervention success; and the acceptability, value and sustainability of the intervention. Interviews were conducted by two researchers (DBW and JH). DBW has extensive experience of conducting qualitative research interviews. Both researchers worked together for the first interview. The remainder were conducted solely by JH, with support and guidance from DBW as required. All interviews were audio recorded and transcribed verbatim for analysis.

### Analysis & Presentation of Data

#### Quantitative data

Simple summaries of the quantitative data are presented in tabular format. No attempts were made to formally test any differences between demonstration and roll-out stores as the intervention models applied differed and the sample of only 17 demonstration stores was not large enough to allow robust analyses.

#### Qualitative data

Qualitative data were analysed using the framework method. [15], [16] This began with familiarisation with transcripts to identify recurrent themes. The first interview transcript was then analysed in-depth, examining each quote and allocating it to a pre-determined or newly emergent theme. The resulting thematic framework was applied to subsequent transcripts and distilled into key subject areas with both representative quotes and summaries of overlapping views.

In the results section, quantitative data related to each specific aim are presented and relevant quotes from the qualitative interviews used to elaborate and develop possible explanations for the quantitative findings. Further themes identified during analysis of the qualitative data are then presented. This approach allows integration of the quantitative and qualitative findings and ensures that each informs interpretation of the other. [17], [18]

### Consent and Research Ethics

Representatives of stores where observations were conducted and individuals who took part in qualitative interviews provided written, informed consent to take part and were offered a £10 high street shopping voucher as a ‘thank you’ for participating. Ethical approval was provided by Newcastle University Research Ethics Committee (application 412).

## Results

All 87 stores were mapped. In-store observations were conducted in 15 of 17 (88%) demonstration stores and 59 of 70 (84%) roll-out stores. Ten interviews were conducted. There were no differences in the proportion of stores that were located in deprived areas, urban areas, areas with poor existing access to FFV, or all three between those stores that did and did not agree to in-store observations (data not shown).

### Fidelity of Intervention Implementation

Table 2 shows the results of the mapping exercise. Most stores were located in areas with poor existing access to FFV. However, only around three-quarters were in either deprived or urban areas. Less than 60% of stores were located in deprived, urban areas with poor existing access to FFV.

**Table 2:** Fidelity of intervention: location.

Marker of fidelity	Demonstration stores (n = 17)	Roll-out stores (n = 70)	All stores (n = 87)
N (%) in most deprived 40% of areas in England	14 (82.4)	53 (75.7)	67 (77.0)
N (%) in urban areas	13 (76.5)	55 (78.6)	68 (78.2)
N (%) >500 m network distance from a supermarket	17 (100)	63 (90.0)	80 (92.0)
N (%) all of above	12 (70.6)	37 (52.9)	49 (56.3)

One retailer who was interviewed felt his store was not in an appropriate location, but that this information was not acted upon (Box S1, quote A).

The prevalence of Change4Life branded point-of-sales materials and equipment was low (less than 40% for all items except Change4Life branded chill cabinet). Similarly, the frequency of appropriate use of these materials and equipment was low (Table 3). Only around half of stores used all of the Change4Life branded point-of-sales materials and equipment present in their stores appropriately.

**Table 3:** Fidelity of intervention: equipment presence & appropriate use.

Marker of fidelity	Demonstration stores (n = 15)	Roll-out stores (n = 59)	All stores (n = 74)
N (%) with Change4Life branded chill cabinet^1^	14 (93.3)	28 (47.5)	42 (56.8)
of which, N (%) used appropriately	9 (64.3)	14 (50.0)	23 (54.8)
N (%) with Change4Life branded shelves	8 (53.3)	19 (32.2)	27 (36.5)
of which, N (%) used appropriately	3 (37.5)	10 (52.6)	13 (48.1)
N(%) with Change4Life branded stand	5 (33.3)	18 (30.5)	23 (31.1)
of which, N (%) used appropriately	0 (0)	6 (33.3)	6 (26.1)
N (%) with fresh fruit & vegetable champion	3 (20.0)	14 (23.7)	17 (23.0)
N (%) with branded chill cabinet, shelves, stand & champion	1 (6.7)	1 (1.7)	2 (2.7)
N (%) with all equipment present used appropriately	5 (33.3)	34 (57.6)	39 (52.7)

1 This refers to chill-cabinet branding only – demonstration stores received a new chill cabinet with Change4Life branding in place. Roll-out stores received stick-on branding materials for any existing chill cabinets.

Those retailers who were provided with part-funding for a chill cabinet identified this as a particularly attractive aspect of the intervention during interviews (Box S1, quote B). Interviewed retailers highlighted that the branded point-of-sales materials were not durable and that replacements were unavailable (Box S1, quote C). Many retailers interviewed felt the programme was intended to be a short-term intervention that had now lost momentum (Box S1, quote D).

The member of the DH strategic leadership team who was interviewed was aware that branded materials were not always used as intended, but believed that there was a policy for preventing this (Box S1, quote E). It is not clear if retailers were aware of this policy, or what penalties could be imposed, given that all materials were provided up-front.

### Variety, Purchase Price and Quality of FFV in Intervention Stores

The variety, quality and comparable purchase price at a major supermarket of FFV in intervention stores is summarised in Table 4. In nine stores, comparable purchase price was available for less than three lines, primarily due to prices not being displayed in stores, and these stores were excluded from the price analyses. The median number of FFV lines per store was 26. Around one in three of these lines fell into ‘core’ categories, defined by intervention guidance as the minimum range that should be stocked (i.e. potatoes, onions, carrots, bananas, apples and tomatoes). Almost all FFV was judged to be of good quality. Overall, FFV in intervention stores cost around 10% more than at www.tesco.com.

**Table 4:** Variety, quality and purchase price of fresh fruit & vegetables.

	Demonstration stores (n = 15)	Roll-out stores (n = 59)	All stores (n = 74)
Median (IQR) number of fresh fruit & vegetables lines	29.0 (23.0–39.0)	24.0 (13.0–33.0)	25.5 (14.8–35.0)
Median (IQR) % of fresh fruit & vegetables lines in ‘core’ categories^1^	32.4 (30.8–35.3)	36.1 (31.2–50.0)	35.0 (31.0–44.4)
Mean (SD) % of fresh fruit & vegetables good quality	99.9 (0.5)	98.9 (4.1)	99.1 (3.6)
Mean (SD) purchase price/item as % of equivalent www.tesco.com purchase price^2^	107.9 (11.9)	109.2 (13.3)^3^	108.9 (12.9)^4^

1 Core categories  =  potatoes, onions, carrots, bananas, apples and tomatoes.

2 restricted to items with comparable line at www.tesco.com.

3 number of stores  = 50, in 9 stores prices were available for fewer than three lines and these were excluded from this analysis.

4 number of stores = 65.

A number of retailers commented during interviews that the intervention prompted them to expand their FFV range (Box S2, quote A). All retailers interviewed seemed conscious of the need to display high quality stock. Spoilage and wastage was identified in interviews as a significant barrier to stocking more FFV. Whilst this problem seemed insurmountable to some retailers, others identified it as a necessary initial stage in expanding their range (Box S2, quote B). Some retailers suggested in interviews that they limited their range of FFV to lines with a longer shelf life for this reason (Box S2, quote C). The issue of waste did not seem to permeate back to the DH team (Box S2, quote D). Many retailers commented that financial support to cover waste, at least in the early stages, would have been welcomed (Box S2, quote E).

### Motivation for Taking Part and Benefits to Retailers

The majority of interviewees were strongly supportive of the health improvement intentions of the intervention (Box S2, quote A). However, the specific aims of the intervention were less clearly understood (Box S4, quote B), and the potential impact on health questioned (Box S6, quote D). Whilst some retailers reported that they took part to support health improvement, most were also motivated by commercial interests. Demonstration store retailers particularly valued part-funding of a chill cabinet, whilst others were glad of free point-of-sale materials. A number of retailers reported that they had little or no choice over whether or not they took part in the intervention and this was associated with lack of engagement (Box S2, quote B).

Whilst the member of the DH strategic leadership team was aware that commercial factors were likely to drive retailer motivations, they did not necessarily view this negatively. In contrast, the health worker interviewed was strongly opposed to DH funding what they felt was a commercial venture (Box S2, quote C).

### Initial and On-going Communication

During interviews it became clear that many retailers and symbol group managers did not feel that there was enough communication between themselves and either the DH strategic leadership team or the project delivery team. Although the member of the DH strategic leadership team stressed that clear communication of the interventions aims to all concerned was central to success (Box S4, quote A), a number of others commented that they were never absolutely clear what the aims were (Box S4, quote B). The loss of momentum of the intervention was frequently blamed by interviewees on poor communication (Box S4, quote C).

### Sustainability Plans and Links with the Public Sector

Linking stores to local health workers, and hence to community initiatives, was not straightforward. In addition to poor communication, this appears to have been largely due to lack of clarity around roles and responsibilities. The health worker interviewed was resistant to taking on a role that they felt was more about business development than health improvement (Box S5, quote A); whilst the regional area manager felt this was exactly the health worker’s role to (Box S5, quote B). Although the health worker, area manager and retailers all gave examples of when they had tried to link up, these were generally stories of failure (Box S5, quote C). The member of the DH strategic leadership team focused more on instances when links between stores and public sector organisations had worked, rather than reasons why, and problems caused, when it had not (Box S5, quote D).

### Effects on Sales, Profit, Diet and Health

Sales of FFV were reported to improve following the intervention. However, this was often against a background of ongoing improving FFV sales (Box S6, quote A). Improvements were also often from a very low base (Box S6, quote B) meaning that any impact on profits was small. Some retailers felt that their small store size meant they would never be able to compete successfully with the major supermarkets on FFV (Box S6, quote C).

Substantial scepticism was expressed during interviews over whether the intervention was effective in improving customers’ diets. Whilst the intervention was particularly targeted at individuals living in more deprived circumstances, this was itself identified as a barrier to success (Box S6, quote D). The health worker wanted to see data from a formal outcome evaluation before drawing conclusions on effectiveness, whilst the member of the DH strategic leadership team described a case study which they felt was strong evidence of effectiveness (Box S6, quote E). There was also some scepticism about the focus of the intervention on fresh, rather than frozen or canned fruit and vegetables – both of which may have been more convenient than FFV for small retailers and consumers (Box S6, quote F).

## Discussion

### Summary of Results

In this process evaluation of the Change4Life convenience store programme in the North East of England, we found substantial evidence that the intervention was unlikely to be effective.

Fidelity in terms of location of intervention stores was relatively high. However, around two years after initial implementation, fidelity in terms of presence and appropriate use of Change4Life branded point-of-sales materials and equipment was low.

A number of possible reasons why the intervention was unlikely to have been successful were identified, including: poor availability of replacement point-of-sale materials; lack of financial support for FFV waste in the early stages; and failure to cement intended links with health workers, schools and community organisations. Poor initial and on-going communication between stakeholders was also identified as a significant problem and likely contributed to the other problems listed.

### Strengths and Weaknesses

This mixed-methods process evaluation sheds light on whether or not the Change4Life convenience store intervention could have led to sustained improvements in customers’ FFV intake from a variety of perspectives. The mapping exercise and observational data give objective information on the fidelity of intervention implementation, whilst the qualitative interviews provide more in-depth understanding. The qualitative component revealed topics of concern that fell outside the quantitative focus and that could have gone unrecognised without the use of mixed-methods. [17], [18]

Our evaluation also represents the only assessment of the long-term implementation of the Change4Life convenience store programme. Although DH did commission a number of small-scale evaluations all were conducted during the early phases of the intervention. [9], [19]

We used an observational proforma designed specifically for this study to collect in-store data. This has not been validated. However, there was evidence of good inter-rater agreement. Given the novel nature of the intervention being evaluated, existing in-store tools [20], [21] would not have met our requirements.

Comparison purchase prices were collected from the major supermarket a maximum of five weeks after in-store observations were conducted. Seasonal price fluctuations may decrease the accuracy of these comparisons. As we collected on-line comparison prices, we were also unable to judge quality of FFV in the major supermarket.

As we did not conduct an outcome evaluation, we are not able to draw any firm conclusions on the effect of the intervention on dietary quality of store customers. Nor did we include any customers in our interviews. The sales increases reported were based on retailer self-reports, rather than objective data. We cannot conclude from this uncontrolled evaluation that the specific components of this intervention were responsible for the changes, particularly sales increases, seen.

### Interpretation of Findings

Whilst the in-store components of the intervention were clear and stable from the start (Table 1), other aspects of the intervention (e.g. links to health workers, schools and community initiatives) never achieved stability. These aspects were added retrospectively and it seems likely that many of the frustrations experienced by interviewees reflected the lack of clarity surrounding these components. Although it is useful to refine interventions as they develop, [22] clear aims, objectives, roles and responsibilities need to be identified early and only altered after dialogue with all parties.

In order to achieve public health benefit, interventions must either have large reach, or large individual level effects, or, a combination of both. [23] The Change4Life convenience store programme had limited reach. In addition, our evaluation suggests that the intervention is likely to have a very low long-term effect size at the individual level, if any at all, because of the low fidelity of the intervention.

### Implication of Findings for Policy, Practice and Research

The tensions caused by introducing the health worker component of the intervention during roll-out suggest that, as far as possible, the nature of any intervention should be clarified and agreed with all parties before widespread implementation. Some early discussions with retailers may also have helped refine the intervention to provide something that was more appropriate for the business environment in which they work.

The need for good and on-going communication between all those involved in funding, planning and delivering complex public health interventions has been discussed extensively. [24], [25] This was clearly recognised by all those we interviewed, but was not always achieved.

The process of identifying intervention stores involved identification of potential stores by the project delivery team in conjunction with regional symbol group managers. The routine statistics that we used in our mapping exercise were not used by the implementation team and this probably explains why not all stores were located in areas that could formally be classified as deprived, urban and with poor existing retail access to FFV. However, the data we used are readily accessible and relatively easy to use. Those delivering interventions should be aware of routine data that can help them during planning phases.

By focusing our evaluation on implementation fidelity and process around two years after initial implementation, we were not able to determine if the intervention ‘worked’ – in terms of increasing FFV intake of store customers. However, we have generated substantial evidence that the intervention was unlikely to have ‘worked’. This study was achieved using limited resources and provides good justification that a larger, more resource intensive, outcome evaluation of this intervention is not warranted. A staged approach to intervention evaluation, as well as intervention development, [26] is likely to represent the most effective use of resources. This should be conducted separately from pilot and feasibility work. [27] Sequencing of these research stages can be guided by evaluability assessment. [23]

## Supporting Information

Box S1 **Illustrative quotes: fidelity of intervention implementation.**
(DOCX)

Box S2 **Illustrative quotes: variety, purchase price and quality of FFV in intervention stores.**
(DOCX)

Box S3 **Illustrative quotes: motivation for taking part and benefits to retailers.**
(DOCX)

Box S4 **Iillustrative quotes: initial and on-going communication.**
(DOCX)

Box S5 **Illustrative quotes: sustainability plans and links with the public sector.**
(DOCX)

Box S6 **Illustrative quotes: effects on sales, profit, diet and health.**
(DOCX)
