# Supplementary material for: The Change4Life Convenience Store Programme to Increase Retail Access to Fresh Fruit and Vegetables: A Mixed Methods Process Evaluation
Source: PLoS One. 2012 Jun 27;7(6):e39431. doi: 10.1371/journal.pone.0039431 (PMC3384642; doi:10.1371/journal.pone.0039431)
Supplement: Box S1 — Illustrative quotes: fidelity of intervention implementation. (DOCX) [file pone.0039431.s001.docx]

1. *“They know we’ve got the fruit shop next door. When I spoke to the guys at [symbol group], I was saying, you know, ‘I’m not going to sell this stuff’....”* (A43; roll-out store retailer; urban, deprived area with poor existing access to fresh fruit & vegetables)
2. *“We were probably able to get a better chill than we would have otherwise have got...I think a lot of the people who’ve piggy backed on the scheme in a parasitic way to get cheap chills”* (A30; demonstration store retailer; rural, not deprived areas with poor existing access to fresh fruit & vegetables)
3. *“It [point-of-sales materials] got tatty, it got damaged, it wasn’t replaced or whatever when we cleaned, and a replacement wasn’t available.”* (B3; symbol group store chain manager)
4. *“Originally we did, the first month or so it [Change4Life mobile stand] was put in a more prominent position....after a while we just moved it back.”* (A3; roll-out store retailer; urban, deprived area with poor existing access to fresh fruit & vegetables)
5. *“We went into one shop and they had a chill cabinet and it was beautifully presented with the Change4Life strips on the shelves, but it was stocked with cold meats and cream cakes and butter...and then we agreed with the steering group, y’know, three strikes and you’re out sort of thing..”* (B4; member of Department of Health strategic leadership team)
