# Supplementary material for: The Change4Life Convenience Store Programme to Increase Retail Access to Fresh Fruit and Vegetables: A Mixed Methods Process Evaluation
Source: PLoS One. 2012 Jun 27;7(6):e39431. doi: 10.1371/journal.pone.0039431 (PMC3384642; doi:10.1371/journal.pone.0039431)
Supplement: Box S2 — Illustrative quotes: variety, purchase price and quality of FFV in intervention stores. (DOCX) [file pone.0039431.s002.docx]

1. *“It’s the same size fixture as its always been but we looked at the range and looked at getting more, other products in so it was a wider range.”* (A79; roll-out store retailer; urban, deprived area with good existing access to fresh fruit & vegetables)
2. *“The barriers that they [retailers] all threw up were well wastage straight away.. It’s worth going through what I call a ‘waste barrier’ where, yes, for a two-three months you probably will get a lot of waste cos people aren’t used to getting fruit and veg off you. But if you don’t keep it fresh nobody will [buy it].”* (B2; symbol group regional area manager)
3. *“There are some things that lend themselves to being fresh like potatoes and swede and cabbage”* (A30; demonstration store retailer; rural, not deprived areas with poor existing access to fresh fruit & vegetables)
4. *“It [waste] didn’t come up as a problem to be honest...I mean it’s one of the things that we were quite surprised about because that was one of the biggest concerns that all the retailers had, was wastage, wastage.”* (B4; member of Department of Health strategic leadership team)
5. *“…you’ve got a very short shelf life on it, and there’s a lot of wastage involved, and there’s a big cost…any waste that we had had to come out of our pocket…It would have been good if there was a contribution to waste actually.”* (B3; symbol group store chain manager)
