# Supplementary material for: The Change4Life Convenience Store Programme to Increase Retail Access to Fresh Fruit and Vegetables: A Mixed Methods Process Evaluation
Source: PLoS One. 2012 Jun 27;7(6):e39431. doi: 10.1371/journal.pone.0039431 (PMC3384642; doi:10.1371/journal.pone.0039431)
Supplement: Box S3 — Illustrative quotes: motivation for taking part and benefits to retailers. (DOCX) [file pone.0039431.s003.docx]

1. *“Obviously there is a lot of fat kids….and it’s not all about you just want to take the money off them on crisps and chocolates and things.”* (A79; roll-out store retailer; urban, deprived area with good existing access to fresh fruit & vegetables)
2. *“Well we had our area manager come initially, like, and come with the stands, like...I wouldn’t say ‘forced upon’...I think he was basically told he had to do it so he pushed it on to me and said I had to do it and that was it.”* (A70; roll-out store retailer; urban, deprived area with poor existing access to fresh fruit & vegetables)
3. *“If the outcome was just to have higher outputs from convenience stores…how can the Department of Health justify that programme?”* (B1; local primary care organisation health worker)
