# Supplementary material for: The Change4Life Convenience Store Programme to Increase Retail Access to Fresh Fruit and Vegetables: A Mixed Methods Process Evaluation
Source: PLoS One. 2012 Jun 27;7(6):e39431. doi: 10.1371/journal.pone.0039431 (PMC3384642; doi:10.1371/journal.pone.0039431)
Supplement: Box S4 — Iillustrative quotes: initial and on-going communication. (DOCX) [file pone.0039431.s004.docx]

1. *“I think it’s about making sure that the retailers fully understand the aims of Change4Life and the change programme....it’s about having clear aims and objectives.”* (B4; member of Department of Health strategic leadership team)
2. *“I guess it might have helped if somebody said ‘well this is why we’re doing it, we want more people to eat more fruit and veg’. And I guess at least then you know exactly what the reason is that you’re doing it.”* (A79; roll-out store retailer; urban, deprived area with good existing access to fresh fruit & vegetables)
3. *“I just think there was maybe a lack of communication about...you know, I’d get an email now and then, but it was rubbish.”* (B3; symbol group store chain manager)
