# Supplementary material for: The Change4Life Convenience Store Programme to Increase Retail Access to Fresh Fruit and Vegetables: A Mixed Methods Process Evaluation
Source: PLoS One. 2012 Jun 27;7(6):e39431. doi: 10.1371/journal.pone.0039431 (PMC3384642; doi:10.1371/journal.pone.0039431)
Supplement: Box S5 — Illustrative quotes: sustainability plans and links with the public sector. (DOCX) [file pone.0039431.s005.docx]

1. *“It’s the kind of thing that if they expect us to go and run their business for them, I think that it’s just not our remit…I wasn’t prepared to hold their hands and strike, y’know, a business deal for them.”* (B1; local primary care organisation health worker)
2. *“They haven’t seen these promised links with the community and links with the doctor. I know the health workers were saying well the shopkeepers aren’t really that keen, but they need to go to the shopkeepers and work with them.”* (B2; symbol group regional area manager)
3. *“Yeah, well it kind of fell apart in that there was supposed to be links with the local school, health workers. For my stores that never occurred.”* (B2; symbol group regional area manager)
4. *“Where we could tie in with the local primary schools and the PCTs [Primary Care Trusts – local primary care organisations] and where that worked well where you had, y’know, an interested head teacher, you had an enthusiastic PCT member of staff and a keen retailer, it worked brilliantly – absolutely brilliantly.”* (B4; member of Department of Health strategic leadership team)
