# Supplementary material for: The Change4Life Convenience Store Programme to Increase Retail Access to Fresh Fruit and Vegetables: A Mixed Methods Process Evaluation
Source: PLoS One. 2012 Jun 27;7(6):e39431. doi: 10.1371/journal.pone.0039431 (PMC3384642; doi:10.1371/journal.pone.0039431)
Supplement: Box S6 — Illustrative quotes: effects on sales, profit, diet and health. (DOCX) [file pone.0039431.s006.docx]

1. *“There’s been a marked increase in fruit and veg sales in the company…We used to do about £2300-2400 a week on fruit and veg. We’re now doing about £5000 a week.”* (B3; symbol group store chain manager)
2. *“…and it has increased, his [demonstration store retailer] fruit and veg sales have gone from about £50 a week now to £150 a week.”* (B2; symbol group regional area manager)
3. *“We’re cheaper than the supermarkets but you know they spend millions on marketing to brainwash you into the fact that they’re doing you a favour.”* (A30; demonstration store retailer; rural, not deprived areas with poor existing access to fresh fruit & vegetables)
4. *“It should benefit the lower demographic, but I don’t think it does...The A’s and B’s, the people who tend to buy fruit and veg anyway, they might just buy a bit more.”* (B3; symbol group store chain manager)
5. *“There was an example we had...a woman...and her and her husband ran a business...and they didn’t have time to cook...and she said she’d been advised by her doctor that she needed to make really big changes to her diet because of her health. The fact that her local store was on the programme and selling fresh fruit and veg made it much easier for her...”* (B4; member of Department of Health strategic leadership team)
6. *“Maybe something is needed to say well yes we want you to eat fruit and veg but there’s nothing wrong with having frozen raspberries in a smoothie”* (A79; roll-out store retailer; urban, deprived area with good existing access to fresh fruit & vegetables)
